# Contact load practices and perceptions in elite English rugby league: an evaluation to inform contact load guidelines

**DOI:** 10.17159/2078-516X/2024/v36i1a17646

**Published:** 2024-08-15

**Authors:** J Parmley, D Weaving, S Whitehead, J Brown, L Fairbank, S Flahive, AJ Gardner, S Hendricks, RD Johnston, P Mackreth, J Peacock, G Phillips, S Scantlebury, J Stein, K Stokes, K Till, B Jones

**Affiliations:** 1 Carnegie Applied Rugby Research (CARR) centre, Carnegie School of Sport, Leeds Beckett University, Leeds, United Kingdom; 2 Department of Physical Activity and Sport, Faculty of Arts and Sciences, Edge Hill University, Ormskirk, United Kingdom; 3 Applied Sports Science and Exercise Testing Laboratory, The University of Newcastle, Ourimbah, NSW, Australia; 4 Institute of Sport and Exercise Medicine (ISEM), Department of Exercise, Sport and Lifestyle Medicine, Faculty of Medicine and Health Sciences, Stellenbosch University; 5 Division of Physiological Sciences and Health through Physical Activity, Lifestyle and Sport Research Centre, Department of Human Biology, Faculty of Health Sciences, University of Cape Town, Cape Town, South Africa; 6 England Performance Unit, Rugby Football League, Manchester, United Kingdom; 7 National Rugby League, Sydney, Australia; 8 Sydney School of Health Sciences, Faculty of Medicine and Health, The University of Sydney, Camperdown, NSW, 2007, Australia; 9 School of Behavioural and Health Sciences, Faculty of Health Sciences, Australian Catholic University, Brisbane, QLD, Australia; 10 Sports Performance, Recovery, Injury & New Technologies (SPRINT) Research Centre, Australian Catholic University, Brisbane, QLD, Australia; 11 Hull Kingston Rovers, Hull, United Kingdom; 12 Centre for Health, and Injury & Illness Prevention in Sport, University of Bath, Bath, UK; 13 Rugby Football Union, Twickenham, UK; 14 Leeds Rhinos Rugby League Club, Leeds, United Kingdom; 15 Premiership Rugby, London, United Kingdom

**Keywords:** rugby league, contact training, load

## Abstract

**Background:**

Athlete exposure to contact could be a risk factor for injury. Governing bodies should provide guidelines preventing overexposure to contact.

**Objectives:**

Describe the current contact load practices and perceptions of contact load requirements within men’s and women’s rugby league to allow the Rugby Football League (RFL) to develop contact load guidelines.

**Methods:**

Participants (n=450 players, n=46 coaching staff, n=32 performance staff, n=23 medical staff) completed an online survey of 27 items, assessing the current contact load practices and perceptions within four categories: “current contact load practices” (n=12 items), “perceptions of required contact load” (n = 6 items), “monitoring of contact load” (n=3 items), and “the relationship between contact load and recovery” (n=6 items).

**Results:**

During men’s Super League pre-season, full contact and controlled contact training was typically undertaken for 15–30 minutes per week, and wrestling training for 15–45 minutes per week. During the in-season, these three training types were all typically undertaken for 15–30 mins per week. In women’s Super League, all training modalities were undertaken for up to 30 minutes per week in the pre- and in-season periods. Both men’s and women’s Super League players and staff perceived 15–30 minutes of full contact training per week was enough to prepare players for the physical demands of rugby league, but a higher duration may be required to prepare for the technical contact demands.

**Conclusion:**

Men’s and women’s Super League clubs currently undertake more contact training during pre-season than in-season, which was planned by coaches and is deemed adequate to prepare players for the demands of rugby league. This study provides data to develop contact load guidelines to improve player welfare whilst not impacting performance.

Rugby league is a collision-based sport involving repeated contact events during matches and training.^[1]^ Players are exposed to contact events in both matches (e.g., repeated ball-carries and tackles) and training (e.g., when preparing for the physical and skill components of contact). The frequency, rate, volume, intensity, and type of exposure to contact during training and matches can be referred to as a player’s ‘contact load’.^[2]^ Considering the high risk of injury and head acceleration exposure during rugby league contact events^[3,4]^ and the potential associations between repetitive head impacts and neurodegenerative disease,^[5]^ contact load exposure guidelines are important for player welfare. World Rugby has published guidelines for rugby union,^[6]^ although no guidelines exist for rugby league.

Contact load is important for performance (e.g., effective tackle performance) and player welfare.^[7]^ The tackle exposure during rugby league match-play has been described;^[1,2]^ however, players spend significantly more time in training than match play, and the contact load of players in training has received limited attention.^[8,9]^ Training is more modifiable compared to match-play, allowing for a more controlled environment with regard to contact, therefore directly impacting the volume and intensity of contact that players are exposed to. Training practices within academy rugby league have been previously described, finding between 8% and 13% of training time dependent on age group and stage of the season was spent on tackle training, with between 10% and 14% of training time utilising small-sided games, which may also include contact.^[10]^ However, this study provided a limited indication of the duration, frequency, and intensity (e.g., full contact at match intensity vs controlled contact) of contact training.

To allow for the development of contact load guidelines, it is necessary to understand the current contact load.^[11]^ Therefore, the current contact load, perceptions of how much is required, how it is monitored, who is responsible for the prescription, and perceptions of the relationship between contact load and recovery require investigation. These findings can inform policy, enabling clubs to train within a framework that has sufficient contact load exposure to ensure players are prepared for match-play while reducing the risk of potential negative outcomes from excessive exposure. This study aimed to describe the current contact load practices and perceptions within men’s and women’s rugby league. Subsequently, the Rugby Football League (RFL), the governing body for rugby league in England, will develop specific contact load guidelines informed by these findings.

## Methods

### Study design

An online survey containing 27 items (Qualtrics, Provo, UT) was designed to assess the current contact load practices and perceptions within both men’s Super League (MSL) and women’s Super League (WSL) clubs (highest standard within RFL competitions). The survey was designed by the authors in consultation with key stakeholders and experts with extensive experience within rugby league to ensure the content of the survey was able to acquire appropriate information regarding contact training practices and perceptions whilst also ensuring the interpretability of questions for the target population. The survey was distributed and completed by players, coaches, performance staff and medical staff.

### Participants

A total of 551 participants completed the survey. This was comprised of 305 from MSL (age=27.4 ± 9.1yrs) including 236 players, 28 coaches, 24 performance staff and 17 medical staff, and 246 from WSL (age=25.7 ± 8.6yrs) including 214 players, 18 coaches, 8 performance staff and 6 medical staff. Respondents represented approximately 55% and 80% of MSL player and staff and 75% and 65% of WSL player and staff populations. Eligibility for participation for the study was described as “Participants must be over the age of 18 years and a player or member of staff working in a men’s or women’s Super League first team. Ethics approval was granted by Leeds Beckett University ethics committee (101700). Participants provided written consent as a prerequisite to completing the survey. All participants were anonymous, and data were stored securely on the online survey platform (Qualtrics, Provo, UT).

### Procedure

The survey was distributed by the RFL via email during the 2023 pre-season. All eligible players and staff were invited to participate via player welfare managers embedded within clubs. The survey characterised participants by club and role. Survey items were multiple-choice questions with ‘unsure’ as an option to improve response accuracy. The survey aimed to assess current contact load practices and perceptions of contact load across 27 items, broadly categorised by four areas: “current contact load practices” (n=12 items), “perceptions of required contact load” (n=6 items), “monitoring of contact load” (n=3 items), and “the relationship between contact load and recovery” (n=6 items). Supplementary 1 Table 1 shows individual survey items. Participants were provided definitions of contact training type, with full contact training being defined as “similar contact intensity as a match”, controlled contact as “similar drills to full contact, but at a reduced intensity and whilst using shields or pads” and wrestling training as “a specific session or any training drill where the particular focus is the wrestling”.

### Statistical analysis

All analyses were completed in R Studio (RStudio version 2022.02.1; R Version 4.2.1). For descriptive purposes, responses to the survey are presented as frequency and percentage per group (players, coaching, performance, and medical staff, and staff overall average) where appropriate.

Exploratory analysis of the construct dimensions within the questionnaire was conducted using exploratory factor analysis (EFA; principal axis method with varimax rotation) using the *psych* package in R Studio.^[12,13]^ Scale and item reliability (internal consistency) were examined using Cronbach’s α using the *psych* package in R Studio.^[14]^ Values above 0.7 were considered acceptable for research purposes, and scores above 0.9 considered excellent internal consistency.^[15]^ Further detail of methods is described in Supplementary 4.

Multiple factor analysis (MFA) using the *FactoMineR* package (version 2.8)^[16]^ was then conducted to assess the agreement in perceptions between players and staff and between players for MSL and WSL, along with individual staffing groups for only MSL due to the number of responses in each staffing group for the four sections of the survey: “current contact load practices”, “perceptions of required contact load”, “monitoring of contact load”, and “the relationship between contact load and recovery”. Escoufier’s Rv coefficient^[17]^ was extracted from the MFA to assess agreement. The R_V_ coefficient is a non-centred squared coefficient of correlation between two matrices. R_V_ coefficient values fall between 0 and 1 and reflect the amount of variance shared by two matrices, with 0 indicating the least shared variance between matrices and 1 indicating the greatest amount of shared variance between matrices.

## Results

### Exploratory factor analysis and internal consistency of questionnaire items

Supplementary 4 Table 4 details the results of the exploratory factor analysis for the four retained factors which captured a total of 41% of the variance of the 25 questionnaire items.

Factor 1 demonstrated strong component loadings for the items evaluating current contact load practices, predominately in pre-season and perceptions of how much time was needed in full contact to prepare players for the physical and technical demands of matches. Factor 2 was represented by perceptions of how contact load relates to recovery, whether the number of matches in a season is appropriate and whether players do an appropriate amount of full contact training to prepare for the physical and technical demands of rugby league. Factor 3 was represented by current contact load practices during the season. Factor 4 was represented by perceptions of how contact load relates to days needed to recover from matches, full- and controlled contact training. The overall Cronbach’s α of the questionnaire was 0.75 suggesting suitable reliability. When each item was removed one at a time, Cronbach’s α remained between 0.71 to 0.76, although reliability increased (α = 0.82) when ‘how contact load is monitored’ was removed.

### Current contact load practices

**Fig. 1:**
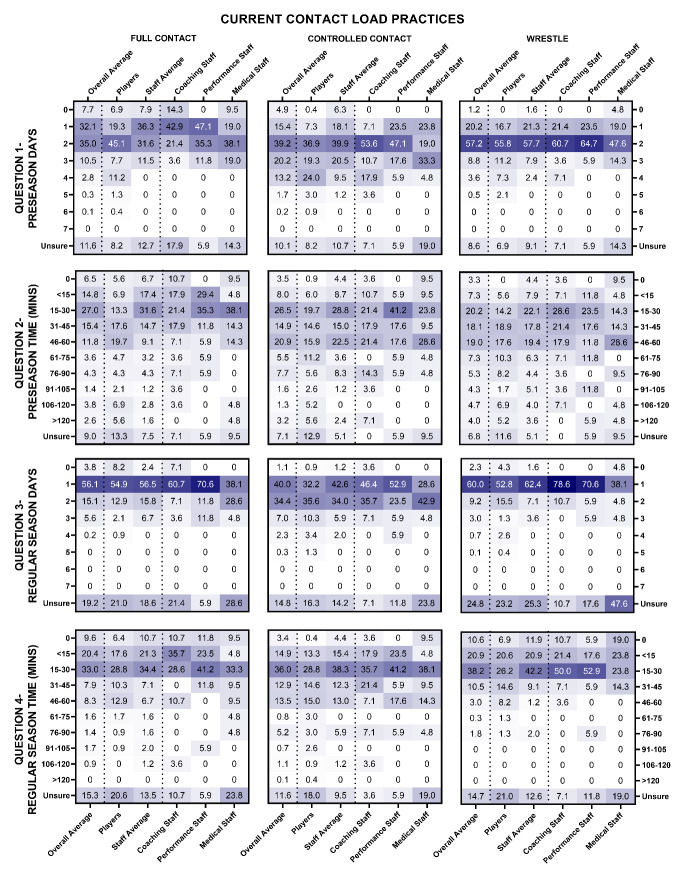
Percentage responses (values) from men’s Super League players and staff (x-axis) on the number of days and duration of time (y-axis) players undertake full contact, controlled contact and wrestling training per week during pre-season (question 1 and 2) and in-season (question 3 and 4). Darker shading representing higher percentage of responses.

**Fig. 2:**
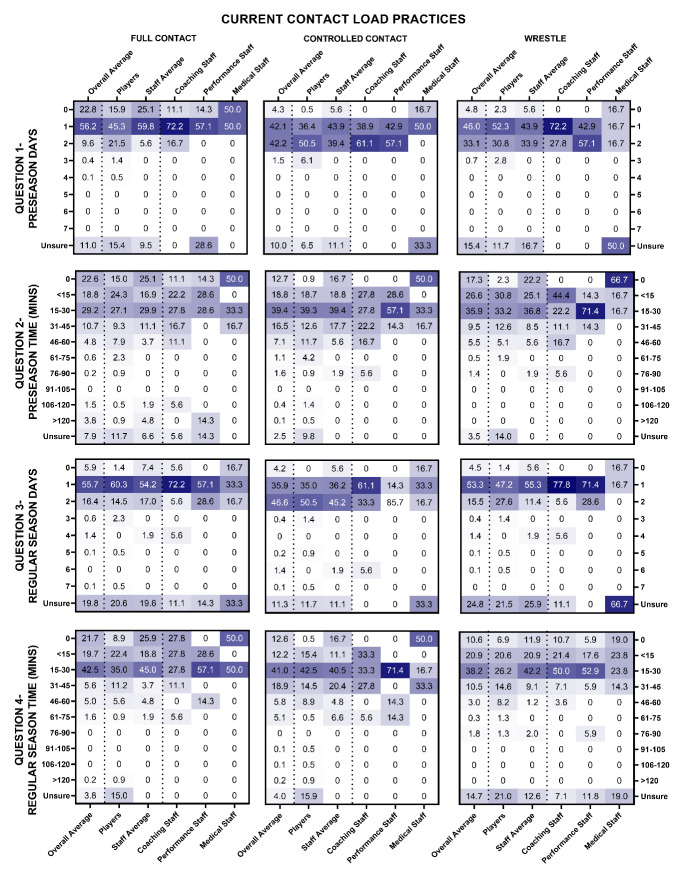
Percentage responses (values) from women’s Super League players and staff (x-axis) on the number of days and duration of time (y-axis) players undertake full contact, controlled contact and wrestling training per week during pre-season (question 1 and 2) and in-season (question 3 and 4). Darker shading represents a higher percentage of responses.

Current contact load practices are shown in Figures 1 and 2 for MSL and WSL.

In MSL during pre-season, full contact training (e.g., similar contact intensity as a match) was typically undertaken for one or two days per week for a total duration of up to 30 minutes. Controlled contact (e.g., similar drills to full contact, but at a reduced intensity and whilst using shields or pads) training was typically undertaken two days per week for a total duration of 15–30 minutes, and wrestling training (e.g., a specific session or any training drill where the particular focus is the wrestling) was typically undertaken two days per week for a total duration ranging from 15–60 minutes per week. During the in-season, full contact, controlled contact and wrestling training were each typically undertaken one day per week for less than 30 minutes total duration (Figure 1).

In WSL during pre-season, full contact training was typically undertaken one day per week for 15–30 minutes, controlled contact training was typically undertaken one or two days for 15–30 minutes in total per week, and wrestling training was typically undertaken one day per week for less than 30 minutes per week. During the in-season, full contact and wrestling training were typically undertaken one day for 15–30 minutes per week. Controlled contact was typically undertaken two days for 15–30 minutes in total per week (Figure 2).

### Perceptions of required contact load

**Fig. 3:**
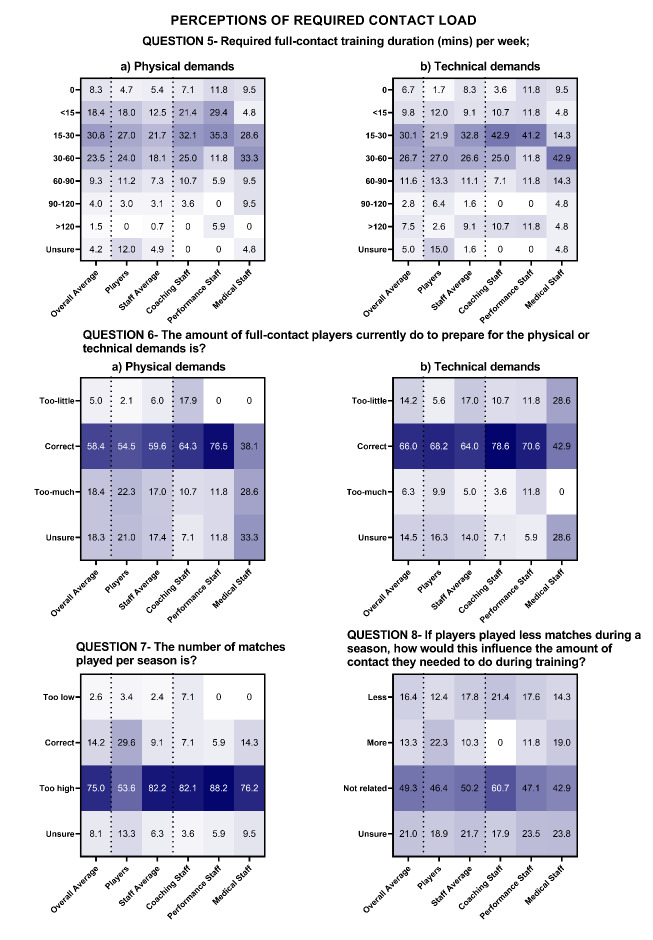
Percentage responses from men’s Super League players and staff on the “perceptions of required contact load” (Question 5–8). Darker shading represents higher percentage of responses.

**Fig. 4:**
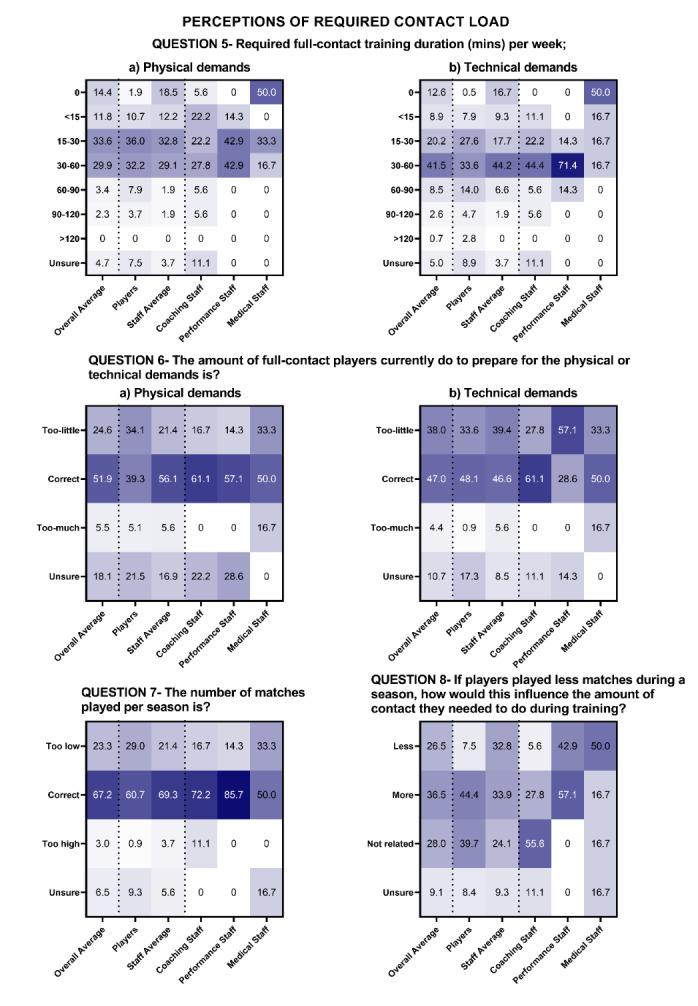
Percentage responses from women’s Super League players and staff on the “perceptions of required contact load” (Question 5–8). Darker shading represents higher percentage of responses.

Staff and player perceptions of how much contact training is required, how this relates to matches, current contact load monitoring practices and perceptions of the relationship between contact load and recovery are shown in Figure 3 and 4 for MSL and WSL.

In MSL, between 15–30 minutes of full contact training was reported as adequate to ensure players are prepared for both the physical and technical demands of rugby league match-play (31% and 30% of respondents; Figure 3, Question 5). The amount of contact that players currently do is adequate to prepare players for the physical and technical demands of match-play (58% and 66% of respondents; Figure 3, Question 6). On average, 75% of respondents reported that the number of matches played throughout the season is too high (Figure 3, Question 7), and the largest proportion of respondents (49.3%) reported that if the number of matches was reduced, the amount of contact training required would not be affected (Figure 3, Question 8).

In WSL, the most common response regarding the required amount of full contact to prepare for the physical demands of rugby league was 15–30 minutes per week (42%) (Figure 4, Question 5a). For the technical demands of rugby league, the amount of contact training required to prepare for the technical demands of rugby league was 30–60 minutes (41%) (Figure 4, Question 5b). The full contact load currently undertaken by players is correct to ensure players are adequately prepared for the physical demands of match play (52% and 47%; Figure 4, Question 6). The number of matches throughout the season did not need to be reduced (Figure 4, Question 7), and if the number of matches were reduced, more contact would be required during training to ensure players are adequately prepared (Figure 4, Question 8).

### Monitoring of contact load

**Fig. 5:**
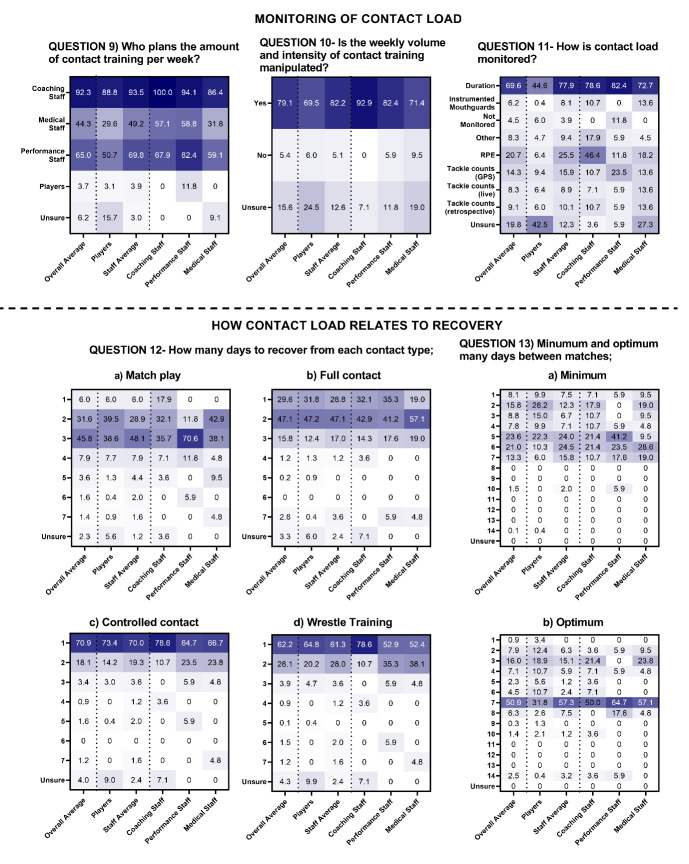
Percentage responses from men’s Super League players and staff on the “monitoring of contact load” (question 9–11) and “how contact load relates to recovery” (question 12 and 13). Darker shading represents higher percentage of responses

**Fig. 6:**
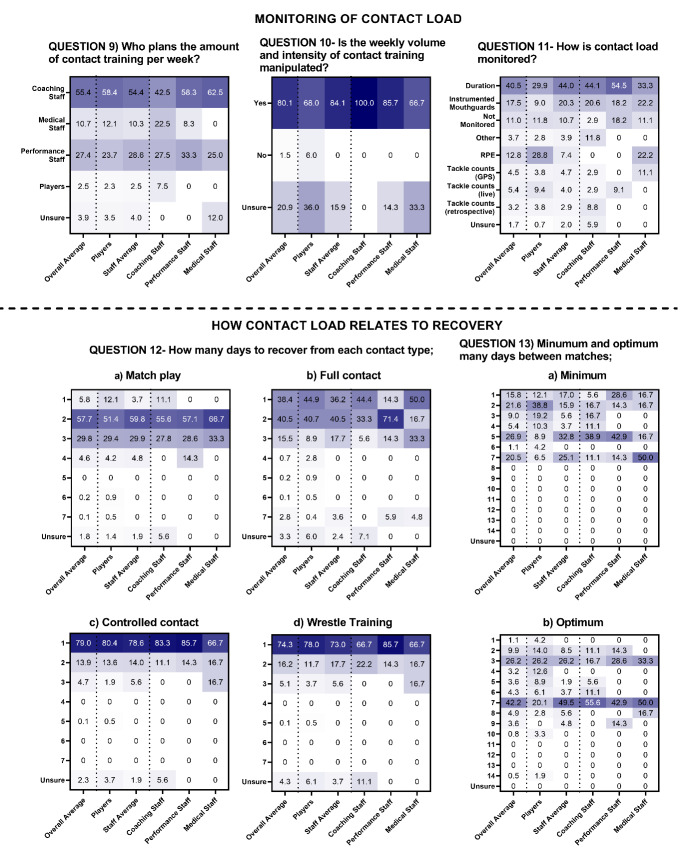
Percentage responses from women’s Super League players and staff on the “monitoring of contact load” (question 9–11) and “how contact load relates to recovery” (question 12 and 13). Darker shading represents higher percentage of responses

In MSL, >90% of players and staff reported that coaches were responsible for planning contact training, that this was manipulated week-to-week (79%), and that contact load was monitored via duration (70%) (Figure 5, Question 11). Similarly, in WSL most players and staff (55.4%) reported that coaches were primarily responsible for planning contact training, that this was manipulated week-to-week (80%) and monitored via duration (41%) (Figure 6).

### How contact load relates to recovery

In MSL, on average, most respondents said it takes players two days to recover fully from full contact training (47.1%), and one day to recover from controlled contact (70.9%) and wrestling training (62.2%). The highest proportion of staff (24% and 24.5%) reported that five to six days was the minimum duration players need to recover between matches, whereas the highest proportion of players (26.2%) reported this to be two days. Most respondents (50.9%) reported that seven days was optimal to recover between matches and adequately prepare for the next match (Figure 5). In WSL, on average, most respondents said it takes players two days to fully recover from full contact training (40.5%), and one day to recover from controlled contact (79%) and wrestling training (74.3%). The highest proportion of staff (32.8%) reported that five days was the minimum duration players need to recover between matches, whereas the highest proportion of players (38.8%) reported two days. Most respondents (42.2%) reported seven days was optimal to recover between matches (Figure 6).

### Comparisons between and within groups (MFA)

The level of agreement between players and staff, along with within-staff group comparisons (coaching, performance and medical staff) are represented using Escoufier’s Rv coefficient (Supplementary 2 Table 2). In MSL the level of agreement between players and staff ranged from Rv = 0.56–0.83, with “monitoring of contact load” and “the relationship between contact load and recovery” showing the lowest agreement, whilst the greatest agreement was shown in “current contact load practices”. Between staff group, comparisons ranged from Rv = 0.35–0.81, with medical staff showing the lowest agreement with other staffing groups. In WSL the agreement in perceptions between players and staff ranged from Rv = 0.50–0.58 with “how contact training relates to recovery” showing the lowest and “monitoring of contact load” showing the greatest agreement.

## Discussion

This study described the perceived contact training practices during preseason and in-season periods within men’s and women’s rugby league to aid the development of specific contact load guidelines. In pre-season, contact training was typically undertaken for one day or two days per week, ranging from 15–45 minutes per week, depending on the training modality. During the in-season, contact training was typically one day or two days per week for a total of 15–30 minutes for all contact training modalities. Fifteen to 30 minutes per week of full contact training was deemed adequate to prepare players for the physical demands of match-play; however, differences existed between players and staff regarding the technical demands. Most players felt they needed to do more full contact training to prepare for the technical demands, compared to staff (30–60 vs 15–30 minutes per week). Coaching staff were generally responsible for planning contact training, and duration was most frequently cited as the method of monitoring contact exposure. In MSL, players and staff believed that it takes two days to recover from full contact training whilst in WSL differences in perception exist (players = one day *vs* staff = two days), however both MSL and WSL participants believe it takes players one day to recover from controlled contact and wrestling training. Overall, in MSL and WSL, it was perceived that the optimum number of days between matches to allow full recovery from contact demands and preparation for the next match was seven days.

### Current contact load practices

The present study is the first to describe the current contact load practices of men’s and women’s rugby league. Previous research in academy male rugby league found Under-19s players undertake tackle training 2.2 ± 0.9 days per week for a total of 86.5 ± 42.7-mins during pre-season and the total volume ranged from 48.5 ± 30.1 to 58.8 ± 37.9-mins in the in-season, dependent on phase (e.g., early, mid, late).^[10]^ This is comparable to the present study with most participants reporting that MSL player’s combined weekly volume of contact training during both preseason and in-season ranges from 45–90 minutes. This is lower than the median combined contact training time in rugby union (100 minutes).^[6]^ The weekly tackle counts during preseason training in academy male rugby league range from 9 ± 5 to 56 ± 39 per week with an average of 10 ± 10 tackles per player per session;^[18]^ however, this may be a blunt measure of contact load as it does not differentiate between contact of different intensities or contact training modality. Understanding the time spent within different contact training modalities is important to add context to how contact load is currently accumulated.

### Perceptions of required contact load

The full contact load guidelines of 30 minutes per week were reported as adequate for players to prepare physically and technically for the contact demands of rugby league match-play in MSL according to the highest proportion of player (36%) and staff (33%) perceptions (Figure 3). In WSL 30 minutes per week was perceived to be adequate for players to prepare for the physical demands of rugby league. However, the perceived required amount to prepare for the technical demands of the game was higher (30–60 minutes).

In MSL, most players (54%) and staff (82%) felt the number of matches per season was too high. In WSL the current number of matches is correct regarding the contributions of match-play to contact load. Differences are likely attributable to the number of matches currently played, with MSL and WSL clubs playing 28–34 and 13–18 matches respectively in the season the survey was completed (Figure 4). Given the number of matches played is a commercial revenue source, it is unclear if players and staff would accept a lower salary for fewer matches. Alternatively, introducing player match limits rather than reducing the number of fixtures per season may achieve this outcome.^[19]^

### Monitoring of contact load

Most respondents stated that coaching staff were responsible for planning contact load in both MSL (92%) and WSL (55%), with performance staff being the second most common response. It is unlikely that players are actively involved in the process of planning and prescribing of contact load, leading to differing perceptions with staff (Rv = 0.56). Medical staff were the staff group least likely to be responsible for the planning of contact load in men (44.3%) and women (10.7%). Similarly, when assessing differences in perceptions within staffing groups, medical staff were least closely aligned to the other staffing groups in their perceptions of contact load (Rv = 0.77). Given the welfare implications of incorrect contact load, it would be prudent for clubs to involve the wider multidisciplinary team, including medical staff, when planning and prescribing contact load, balancing performance, and player welfare.

### How contact load relates to recovery

The highest proportion of respondents reported it takes two days to recover from full contact training in MSL and WSL. The highest proportion of respondents from MSL and WSL said it takes three days and two days to recover from match-play, similar to research showing neuromuscular fatigue, perceptions of fatigue, and muscle soreness remain elevated 48-hours post-match.^[20]^ Most respondents felt that seven days was the optimum timeframe to recover physically from the previous match and allow players to prepare for the next match (Figure 5 and 6). There was a lack of agreement about how contact load relates to the recovery between players and staff in MSL and WSL and between staffing groups in MSL, indicating the need for further objective analysis of contact load and recovery within rugby league.

### Policy implications: contact load guidelines for men’s and women’s Super League

The findings of this study have contributed to informing the development of RFL contact load guidelines, effective from January 2024.

- During pre-season, full contact training should not exceed a total duration of 30 minutes per week; controlled contact should not exceed a total duration of 30 minutes per week; and wrestling training should not exceed a total duration of 45 minutes per week.
- During the regular season, full contact, controlled contact and wrestling training should each not exceed 30 minutes per week.
- Full contact training should not be undertaken on the day before a match or in the two days following a match (where the player participates in more than 20 minutes of a match).
- In Men’s Super League it has been recommended the number of matches should be reduced from the current 28–34 matches across a season, whilst remaining the same in Women’s Super League (13–18 matches).
- Where possible, matches should be played on a seven-day turnaround, with a minimum turnaround between matches of five days. Where a five-day turnaround takes place, a longer rest period should follow.
- Full contact, controlled contact, and wrestling training should be accurately monitored via duration and using instrumented mouthguards where possible.

Using the most common responses by players and staff within each club, the extent to which these guidelines would see a reduction in contact load is shown in Supplementary 3 Table 3. For example, in MSL, based on players ‘ perceptions, the full and controlled contact load during pre-season would be reduced by up to 58%, and wrestling training reduced for 50% of clubs.

During the in-season period, 17% of clubs would reduce full contact and controlled contact load, and wrestling training would be reduced by 8%. In WSL, based on players’ perceptions contact load would be reduced for 8% of clubs for full contact and controlled contact in pre-season, with no change to pre-season wrestling training. During the in-season period, no change in full contact training would occur, however 8% of clubs would see a reduction in controlled contact and wrestling training. Whereas according to staff perception in pre-season, full contact training time would be reduced for 33% of clubs, controlled contact would be reduced for 42% of clubs, and 17% would see a reduction in wrestling training.

Guidelines regarding the scheduling of full contact training and matches have also been developed. For example, full contact training should not occur less than one day before and two days following a match to support player recovery. Based on player and staff perceptions, it has been recommended that the current number of matches in the MSL season (28–34 matches) be reduced. This may be a Super League-specific recommendation, rather than rugby league more broadly (e.g., Australian NRL play 24 matches per season).

The rugby league guidelines allow clubs a greater duration of time during the week to undertake full contact training compared to World Rugby (30 *vs* 15 minutes per week).^[6]^ However, rugby league guidelines have a reduced duration of time for controlled contact (30 minutes *vs* 40 minutes). Wrestling training was included in place of rugby union’s live set piece guidelines, allowing for 45 and 30 minutes in preseason and in-season periods, respectively. The inherent differences between the technical and physical demands of rugby league and rugby union, mean direct comparisons are not possible.^[2,6,21]^

### Limitations

The primary limitation of this study is the use of subjective data to present contact load, which may be affected by many factors.^[22]^ The lack of agreement between groups shows future research should focus on objective analysis of contact load. Using a survey may provide ambiguity and misunderstanding of certain terms and questions and a difference in understanding between participants. The survey was developed in consultation with experts, and exploratory factor analysis appeared to suggest that the survey design and question groupings were adequate, along with this the survey contained suitable internal consistency. Despite this there was no test-retest reliability undertaken. Therefore, the reproducibility of results and stability of perceptions over time is unknown. It is also important to note that on an individual level, players and positional groups may complete different types of contact training (e.g., individual contact skill development). This is likely to result in variation in the magnitude and frequency of collision events within a given duration of contact training. The impact of the recommendations might differ on an individual and positional level given that forwards are involved in a greater number of contact events during match play compared to backs; ^[1]^ therefore, players should be managed individually alongside these guidelines. Future research should focus on using objective data to understand the frequency and magnitude of collision events. Finally, whilst the findings of this study informed RFL contact load guidelines, the efficacy or effectiveness of these guidelines from both performance and welfare perspectives have not been evaluated.

## Conclusion

Overall, based on the highest proportion of responses, clubs in MSL and WSL currently undertake between 15–45 minutes of contact training dependent on modality during pre-season and between 15–30 minutes during the in-season. Players and staff typically feel this is appropriate to adequately prepare players for the physical and technical demands of rugby league. Coaching staff are most likely to plan contact load, and it is primarily monitored by session duration. It is perceived that it takes players two days to recover from full contact training and that seven days between matches is optimal to allow for recovery from match contact demands and allow sufficient preparation for the next match. It is important to note that differences in perceptions exist between players and staff along with within-staff groups, therefore, objective data are needed, in addition to the investigation of the efficacy or effectiveness of RFL guidelines from a performance and injury perspective for MSL and WSL players.

## Supplementary Information
