# Supplementary material for: Contact load practices and perceptions in elite English rugby league: an evaluation to inform contact load guidelines
Source: S Afr J Sports Med. 2024 Aug 15;36(1):v36i1a17646. doi: 10.17159/2078-516X/2024/v36i1a17646 (PMC11374316; doi:10.17159/2078-516X/2024/v36i1a17646)
Supplement: Supplementary file 1 [file 2078-516X-36-v36i1a17646-s001.pdf]

Contact load practices and perceptions in elite English rugby league: an evaluation to inform contact load guidelines

Supplementary 1: Table 1

Detail of individual question items

**Current contact load practices**
1) During pre-season how many days do you do contact training per week; (a) full-contact (e.g., similar contact intensity as a match), (b) controlled-contact e.g., similar drills to full contact, but at a reduced intensity and whilst using shields or pads, (c) wrestle training (a specific session or any training drill where the particular focus is the wrestle)?
2) During pre-season how much contact training do you typically do per week? (minutes) (a) full-contact (e.g., similar contact intensity as a match), (b) controlled-contact e.g., similar drills to full contact, but at a reduced intensity and whilst using shields or pads, (c) wrestle training (a specific session or any training drill where the particular focus is the wrestle)?
3) During the regular season (e.g., in-season when have weekly matches) how many days do you typically do contact training per week; (a) full-contact (e.g., similar contact intensity as a match), (b) controlled-contact e.g., similar drills to full contact, but at a reduced intensity and whilst using shields or pads, (c) wrestle training (a specific session or any training drill where the particular focus is the wrestle)?
4) During the regular season (e.g., in-season when you have weekly matches) how much contact training do you typically do per week; (a) full-contact (e.g., similar contact intensity as a match), (b) controlled-contact e.g., similar drills to full contact, but at a reduced intensity and whilst using shields or pads, (c) wrestle training (a specific session or any training drill where the particular focus is the wrestle)?
**Perceptions of required contact load**
5) How much full-contact (e.g., same intensity as a match) training per week do you think you need to do to prepare players for the (a) physical and (b) technical demands of rugby league? (mins)
6) Do you think players do too-much, too-little or the correct amount of full-contact (e.g., same intensity as a match) training per week to prepare for the (a) physical and (b) technical demands of rugby league?
7) Do you think the number of matches players are required to play in a season is too low, correct, too high, unsure?
8) If players played less matches during a season, how would this influence the amount of contact they needed to do during training?
**Monitoring of contact load**
9) Who plans the amount of contact training per week? (select multiple)
10) Is the weekly volume and intensity of contact training manipulated (e.g., easy, moderate, hard weeks)?
11) How is the amount of contact training players are exposed to per week monitored? (select multiple)
**How contact load relates to recovery**
12) How many days does it take players to physically recover (e.g., no muscle soreness or reduced physical performance) from each contact type; (a) match play, (b) full-contact (e.g., similar contact intensity as a match), (c) controlled-contact e.g., similar drills to full contact, but at a reduced intensity and whilst using shields or pads, (d) wrestle training (a specific session or any training drill where the particular focus is the wrestle)?
13) What are the (a) minimum and (b) optimum number of days between matches to allow players to fully recover from the contact demands and prepare for the next match?

Supplementary 2: Table 2

Escoufier's Rv coefficient derived from MFA, assessing the agreement in perceptions of contact load between Player's and staff for Men and Women's Super League along with between staff agreement for Men's Super League. RV coefficient values fall between 0 and 1 and reflect the amount of variance shared by two matrices.

**Current contact load practices**		
	**Men**	**Women**
Players *vs.* Staff	0.83	0.55
Coaching Staff *vs.* Performance Staff	0.81	N/A
Coaching Staff *vs.* Medical Staff	0.77	N/A
Performance Staff *vs.* Medical Staff	0.79	N/A
**Perceptions of required contact load**		
	**Men**	**Women**
Players *vs.* Staff	0.73	0.52
Coaching Staff *vs.* Performance Staff	0.6	N/A
Coaching Staff *vs.* Medical Staff	0.64	N/A
Performance Staff *vs.* Medical Staff	0.58	N/A
**Monitoring of contact load**		
	**Men**	**Women**
Players *vs.* Staff	0.56	0.58
Coaching Staff *vs.* Performance Staff	0.79	N/A
Coaching Staff *vs.* Medical Staff	0.81	N/A
Performance Staff *vs.* Medical Staff	0.72	N/A
**How contact load relates to recovery**		
	**Men**	**Women**
Players *vs.* Staff	0.56	0.50
Coaching Staff *vs.* Performance Staff	0.74	N/A
Coaching Staff *vs.* Medical Staff	0.35	N/A
Performance Staff *vs.* Medical Staff	0.47	N/A

Supplementary 3: Table 3

The percentage of clubs that would see a reduction in contact load based on proposed guidelines.

**Men's Super League**			
**Pre-season**			
**Training Type**	**Guideline**	**Reduction in contact load for Clubs (%)**	
		**Players' perception**	**Staff perception**
Full-contact	should not exceed a total duration of 30 minutes per week	58%	8%
Controlled-contact	should not exceed a total duration of 30 minutes per week	58%	33%
Wrestle Training	should not exceed a total duration of 45 minutes per week	50%	33%
**Regular Season**			
**Training Type**	**Guideline**	**Reduction in contact load for Clubs (%)**	
		**Players' perception**	**Staff perception**
Full-contact	should not exceed a total duration of 30 minutes per week	17%	17%
Controlled-contact	should not exceed a total duration of 30 minutes per week	17%	0%
Wrestle Training	should not exceed a total duration of 30 minutes per week	8%	0%
**Women's Super League**			
**Pre-season**			
**Training Type**	**Guideline**	**Reduction in contact load for Clubs (%)**	
		**Players' perception**	**Staff perception**
Full-contact	should not exceed a total duration of 30 minutes per week	8%	33%
Controlled-contact	should not exceed a total duration of 30 minutes per week	8%	42%
Wrestle Training	should not exceed a total duration of 45 minutes per week	0%	17%
**Regular Season**			
**Training Type**	**Guideline**	**Reduction in contact load for Clubs (%)**	
		**Players' perception**	**Staff perception**
Full-contact	should not exceed a total duration of 30 minutes per week	0%	8%
Controlled-contact	should not exceed a total duration of 30 minutes per week	8%	25%
Wrestle Training	should not exceed a total duration of 30 minutes per week	8%	8%

Supplementary 4

Exploratory factor analysis (EFA)

EFA requires a complete data matrix. Missing data was evaluated, with missing data ranging from 0 to 9% for each item (mean ± standard deviation = 3 ± 2%). Missing data per participant ranged from 0 to 7 items and therefore data was considered missing at random. To avoid excluding participants, missing data was imputed using the missForest package (1) which uses a non-parametric imputation method to handle mixed data types using a random forest. Prior to EFA, assumptions of suitability of the matrix were determined via Kaiser-Meyer-Olkin (KMO) measure for sampling adequacy (values greater than 0.5 is acceptable) and Bartlett's test of sphericity to ensure that the correlation matrix was not random. The number of factors to retain for interpretation was completed using several criteria, including retaining factors with an eigenvalue greater than 1, visual examination of scree plot, and parallel analysis alongside underlying theory of the underlying factors examined (i.e., "current contact load practices", "perceptions of required contact load", "monitoring of contact load" "the relationship between contact load and recovery"). Based on sample size, a factor loading of individual items of at least 0.3 was considered a significant contributor to the factor structure (2) whilst factors were considered appropriate if they had at least three loadings greater than 0.3.

Exploratory factor analysis and internal consistency of questionnaire items

Bartlett's test of sphericity suggested that the correlation matrix was not random ($X^2 = 6351.782$; $p < 0.001$). Overall KMO was 0.77 with individual items ranging from 0.55 to 0.88 (KMO: 0.5 to 0.6 [n = 2 items]; 0.6 to 0.7 [n = 4 items]; 0.7 to 0.8 [n = 11 items]; > 0.8 [n = 8 items]) suggesting suitable sampling adequacy. Methods (eigenvalues, parallel analysis, scree plot) to determine suitable number of factors to retain indicated 4 factors to retain (Supplementary Table 4).

Supplementary 4: Table 4

Results of the exploratory factor analysis including eigenvalues, percentage of variation explained by each factor and the item loadings for each factor. Loadings greater than 0.4 are bolded, whilst those lower than 0.4 are reported for transparency but with font minimised.

	Factor 1	Factor 2	Factor 3	Factor 4
Eigenvalues	5.3	2.98	1.91	1.28
% of variance explained	16%	9%	9%	8%
**Item Loadings**				
Pre-season Days: Full Contact	**0.56**	0.19	0.11	0.11
Pre-season Days: Controlled Contact	0.35	**0.51**	-0.09	0.28
Pre-season Days: Wrestle	0.29	**0.46**	-0.14	0.37
Pre-season Time: Full Contact	**0.74**	0.2	0.09	-0.05
Pre-season Time: Controlled Contact	**0.7**	0.34	-0.16	0.09
Pre-season Time: Wrestle	**0.61**	**0.45**	-0.1	0.01
Regular Season Days: Full Contact	0.29	-0.15	0.22	**0.43**
Regular Season Days: Controlled Contact	0.25	0.05	-0.03	**0.7**
Regular Season Days: Wrestle	0.05	-0.03	0.07	**0.88**
Regular Season Time: Full Contact	**0.76**	-0.13	0.1	0.18
Regular Season Time: Controlled Contact	**0.67**	0.1	-0.13	0.28
Regular Season Time: Wrestle	**0.55**	0	-0.06	**0.42**
How Much Full Contact: Physical Demands	**0.66**	-0.26	-0.02	0.17
How Much Full Contact: Technical Demands	**0.66**	-0.13	-0.01	0.11
The amount of contact: Physical Demands	0.16	**0.44**	0.19	-0.1
The amount of contact: Technical Demands	0.02	0.37	0.13	0.02
Number of matches	0.15	**0.61**	0.11	-0.1
If less matches were played	-0.07	-0.1	-0.08	-0.11
Who plans the contact training?	-0.07	0.13	-0.15	0.03
Is contact training manipulated?	0.08	0.02	-0.11	0.05
How is contact training monitored?	-0.03	-0.08	0.07	-0.04
How many days to recovery from: Matchplay	-0.05	**0.41**	**0.49**	-0.02
How many days to recovery from: Full Contact	0.03	0.26	**0.75**	0.04
How many days to recovery from: Controlled Contact	-0.02	0.13	**0.85**	0.18
How many days to recovery from: Wrestle	0.04	0.18	**0.81**	0.16
Minimum days to recover: Matchplay	-0.09	**0.52**	0.11	0
Optimum days to recover: Matchplay	-0.09	**0.46**	-0.01	0.01

1. Stekhoven DJ. missForest: Nonparametric Missing Value Imputation using Random Forest [Internet]. 2022 [cited 2024 Mar 17]. Available from: https://cran.r-project.org/web/packages/missForest/index.html. 2. Hair JF. Multivariate Data Analysis. Prentice Hall; 1998. 730 p.
